# Lymphatic marker podoplanin/D2-40 in human advanced cirrhotic liver- Re-evaluations of microlymphatic abnormalities

**DOI:** 10.1186/1471-230X-10-131

**Published:** 2010-11-08

**Authors:** Hiroaki Yokomori, Masaya Oda, Fumihiko Kaneko, Shigeyuki Kawachi, Minoru Tanabe, Kazunori Yoshimura, Yuko Kitagawa, Toshifumi Hibi

**Affiliations:** 1 Division of Gastroenterology of Internal Medicine, Kitasato Medical Center Hospital, Kitasato University, Saitama, Japan; 2 Organized Center of Clinical Medicine, International University of Health and Welfare, Tokyo, Japan; 3 Department of Surgery, Internal Medicine, School of Medicine, Keio University, Tokyo, Japan; 4 Division of Gastroenterology and Hepatology, Internal Medicine, School of Medicine, Keio University, Tokyo; 5 Department of Rehabilitation, Nihon Institute of Medical Science, Saitama, Japan

## Abstract

**Background:**

From the morphological appearance, it was impossible to distinguish terminal portal venules from small lymphatic vessels in the portal tract even using histochemical microscopic techniques. Recently, D2-40 was found to be expressed at a high level in lymphatic endothelial cells (LECs). This study was undertaken to elucidate hepatic lymphatic vessels during progression of cirrhosis by examining the expression of D2-40 in LECs.

**Methods:**

Surgical wedge biopsy specimens were obtained from non-cirrhotic portions of human livers (normal control) and from cirrhotic livers (LC) (Child A-LC and Child C-LC). Immunohistochemical (IHC), Western blot, and immunoelectron microscopic studies were conducted using D2-40 as markers for lymphatic vessels, as well as CD34 for capillary blood vessels.

**Results:**

Imunostaining of D2-40 produced a strong reaction in lymphatic vessels only, especially in Child C-LC. It was possible to distinguish the portal venules from the small lymphatic vessels using D-40. Immunoelectron microscopy revealed strong D2-40 expression along the luminal and abluminal portions of the cell membrane of LECs in Child C-LC tissue.

**Conclusion:**

It is possible to distinguish portal venules from small lymphatic vessels using D2-40 as marker. D2-40- labeling in lymphatic capillary endothelial cells is related to the degree of fibrosis in cirrhotic liver.

## Background

During cirrhosis, the hepatic microvascular phenotype is transformed from sinusoids to continuous capillaries [1]. This crucial process includes excessive deposition of extracellular matrix in the space of Disse. Eventually, formation of basement membranes and defenestration of endothelial cells occur, thereby compromising the normal transfer of nutrients between sinusoidal blood and hepatocytes. All metabolites exchanged between the bloodstream and the hepatocytes pass through the space of Disse that extends from the nonluminal side of the sinusoidal endothelial cell to the microvilli of the hepatocellular membrane, and is considered to be confluent with the hepatic lymphatics of the portal tracts [2]. Increased lymph flow is known to occur in cases of diffuse abnormalities of the liver architecture, e.g. fibrosis and cirrhosis [3]. In human cirrhosis, increased lymph production has been described repeatedly; dilated lymph vessels were documented using angiography and computer tomographic scans in liver fibrotic and cirrhotic patients [4].

Hepatic lymph vessel expansion and its functional relation with blood hepatocytic exchange capacity have been studied *in vivo *by fluorescence microscopy in animal models of fibrosis [5]. Morphologically, liver tissue from human patients with cirrhosis or obstructive jaundice has been analyzed thoroughly using transmission and scanning electron microscopy, and dilatation of the lymphatic vessels has been reported [6]. Lymphatic vessels on the liver surface have also been observed macroscopically during laparoscopy, and dilatation of these superficial lymph vessels during the course of several diseases has also been reported [7].

Blood vessels are identifiable using several immunohistochemical methods. Immunostaining for α smooth muscle actin, factor VIII-associated antigen, and alkaline phosphatase (ALPase) are strongly positive in blood vessels but negative or only weakly positive in lymphatic vessels [8]. These staining methods have therefore been used to differentiate between blood and lymphatic vessels. In addition, 5-nucleotidase activity has been reported to be higher in lymphatic vessels than in blood vessels. A quantitative analysis of lymph vessels using 5-nucleotidase showed marked increases in lymph vessel density and area in rat liver tissues affected by fibrosis and cirrhosis [9]. Using the methods described above, the numbers and areas of lymphatics were found to differ significantly depending on the degree of liver cirrhosis, but were not affected by the activity of hepatitis [10]. Superficial lymphatic vessels have been reported to be more clearly visible in cirrhotic liver [10].

Lymphatic vasculature has recently emerged as a prominent area of research, especially in the identification of lymphatic endothelial specific markers and regulators such as vascular endothelial growth factor receptor (VEGFR)-3, vascular endothelial growth factor (VEGF)-C/D, homeobox prospero-like protein (PROX1), D2-40, lymphatic vessel endothelial hyaluronan receptor-1 (LYVE-1), ephrinB2, and Forkhead Box C2 (FOXC2). Furthermore, development of mouse models has laid the foundation that supports our understanding of the major steps controlling growth and remodeling of lymphatic vessels [11].

A widely used marker of lymphatic endothelial cells in both normal and tumor tissues is LYVE-1, a homolog of hyaluronan receptor CD44 [12]. LYVE-1 is expressed on luminal and abluminal surfaces of lymphatic endothelium, and on hepatic sinusoidal endothelial cells [13]. LYVE-1 expression is attenuated in cirrhotic nodules and absent in HCC liver compared to the normal sinusoidal counterpart [13]. The monoclonal antibody D2-40 raised against a MW 40 kD membrane sialomucin stained the endothelium of lymph vessels but did not react with the endothelium of capillaries, arteries, and veins in normal and neoplastic formalin-fixed paraffin-embedded tissues [13]. Furthermore, D2-40 has been shown to react with glycosylated and non-glycosylated epitopes of gp36. This molecule is also identical to podoplanin [14]. CD34 is a type 1 transmembrane sialomucin mostly present on vascular endothelium in the liver but has been reported on lymphatics in some studies. CD34 is absent from most sinusoidal endothelial cells in normal liver but expression increases during capillarisation in chronic inflammatory disease and in the sinusoidal-type vasculature within hepatocellular carcinomas [15]. Sinusoidal capillarization indicated by CD34 expression correlated with dedifferentiation of the liver tissue during the course of cirrhosis [16].

In fact, D2-40 and podoplanin have been demonstrated as the most sensitive and specific markers for lymph vessel endothelium. The present study was undertaken to elucidate human hepatic lymphatic vessels using D2-40 antibodies during the course of cirrhosis progression, and to investigate the relation with liver cirrhosis. Moreover, blood vessels were also investigated using CD34 antibodies mainly as the blood vessel endothelial markers.

## Methods

### Materials

Surgical liver samples were collected at the Kitasato Institute medical Center Hospital, Kitasato University and School of Medicine, Keio University between 1997 and 2002. As controls, wedge biopsy specimens from normal portions of the liver were obtained from six patients (five male and one female; aged 63-72 years, mean 67.9 years) who underwent surgical resection for metastatic liver carcinoma (all colonic carcinomas). Cirrhotic liver specimens were obtained from a group of five patients (4 males and 1 female; aged 64-74 years, mean 67.4 years) with hepatocellular carcinoma (HCC) and hepatitis C-related cirrhosis of Child-Pugh grade A [17], who underwent hepatectomy (Child A-LC group). Specimens were obtained from the gross cirrhotic portions resected surgically. All patients were evaluated before operation using the indocyanine green (ICG) clearance test, and all had ICG less than15%. Cirrhotic liver specimens were obtained from another group of five patients (five males; aged 58-65 years, mean 61.2 years) with HCC and cirrhosis of Child-Pugh grade C, who underwent liver transplantation (Child C-LC group). All patients in this group had three or fewer tumor nodules with maximal diameter not exceeding 5 cm, but no signs of vascular invasion [18].

### Ethical Approval

The study protocols were approved by the Ethics Committee of the Kitasato Institute Medical Center Hospital, Kitasato University.

### Immunohistochemistry

Mouse monoclonal antibody D2-40 (Signet Laboratories, Inc., Dedham, MA) was used at a dilution of 1:200, and the streptavidin-biotin immunoperoxidase method was used for detecting immune complexes. The sections were deparaffinized and rehydrated. Then sections for D2-40 detection were autoclaved for 7 min at 120°C in 10 mM citrate buffer solution (pH 6.0). After blocking endogenous peroxidase with 0.03% hydrogen peroxide, the sections were incubated with D2-40 antibodies overnight at 4°C. The sections were then incubated at room temperature for 30 min with goat anti-mouse immunoglobulin conjugated to a peroxidase-labeled amino acid polymer, as provided in the SAB-PO (M) Kit (Nichirei Corp., Tokyo, Japan). The sections were counterstained with hematoxylin.

Combined use of lymphatic vessel endothelial markers with pan-endothelial markers, such as D2-40 with CD34, is a practical method to distinguish between blood vessels and lymphatic vessels [19,20]. Therefore, we immunostained sequential sections with antibodies against CD34 for immunohistochemical expression of blood vessel endothelial cell markers. Sections were treated with primary antibodies against CD34 (Dako) for 30 min at room temperature (RT) and subsequently developed using the Envision system (Dako). Bound antibodies were visualized by peroxidase reaction in a 3,3'-diaminobenzoic tetrahydrochloride (DAB) and H_2_O_2 _solution.

### Computer-assisted morphometric analysis of the lymphatic and vascular network

Morphometric variables were determined by labeling lymphatic vessels with anti-human D2-40 and blood vessels with anti-CD34 antibody. Three different portal regions and regenerative fibrotic areas were assessed separately for each section of cirrhotic liver. Briefly, the immunostained sections were scanned using a light microscope at low magnification (×40) and vessels were counted using high magnification (×200; 0.304 mm^2^/field) in each of the regions. Image analysis was done using a computer. Images were captured with the microscope and digitalized using an internal frame-grabbing board (Photoshop CS4/Photoshop CS4 Extended; Adobe Systems, Inc.). This procedure consisted of converting the captured image in points or pixels according to the red tone to assess the lymphatic capillary vessel density (LCVD) or capillary blood vessel density (CBVD). The number of pixels per square millimeter was derived from image analysis [21].

Differences in vessel density were analyzed using one-way ANOVA. A post-hoc Fisher's analysis was performed on statistically significant effects to detect differences between the groups.

### Immunogold-silver staining method for electron microscopy

Liver tissues were fixed in periodate-lysine-parafomaldehyde (PLP). Semi-thin 5 μm sections were immersed for 15 min in three changes of 0.01% phosphate buffered saline (PBS; pH 7.4). Then the sections were incubated with mouse anti-D2-40 monoclonal antibody (Signet) diluted 1:40 in 0.01 M phosphate buffered saline containing 1% bovine serum albumin, overnight at 4°C in a moisture chamber. After rinsing for 15 min in PBS three times, the sections were incubated in 1.4 nm colloidal gold-conjugated IgG antibody (Nanoprobes Inc., Yaphank, NY) diluted 1:50 for 40 min. Finally, the sections were physically developed using a silver enhancement kit (Nanoprobe Silver Enhancement Kit; Nanoprobes) [22]. For transmission electron microscopy, the materials were treated for 15 min PBS three times and fixed in 1.2% glutaraldehyde buffered with 0.01% phosphate buffer (pH 7.4) for 1 h at 4°C, followed by a graded series of ethanol solutions and postfixation with 1% osmium tetroxide in 0.01% phosphate buffer (pH 7.4). After embedding in Epon, ultrathin sections were cut with a diamond knife on an LKB ultramicrotome. They were then stained with uranyl acetate and observed under a transmission electron microscope (JEM-1200 EX; JEOL Tokyo, Japan) with 80-kV acceleration voltage.

The immunogold labeling of lymphatic endothelial cells in the ultrathin sections was quantitated using the Mac Measure software (NIH Image ver. 1.62). Lymphatic endothelial cells around portal tracts (×4000) were selected randomly. The gold particles per unit length of membrane were counted (*n *= 12). Statistical significance of the difference between control and cirrhotic samples was assessed using Student's *t*-test, and *p *< 0.05 was inferred as significant. Data are expressed as mean ± SEM.

### Western blotting

Tissue samples were homogenized in 10 volumes of homogenization buffer (20 mM Tris-HCl, pH 7.5, 5 mM MgCl_2_, 0.1 mM PMSF, 20 μM pepstatin A, and 20 μM leupeptin) using a polytron homogenizer at setting 7 for 90 s. The membrane proteins thus obtained were used for immunoblotting. Proteins (20 ng/ml) were separated using SDS/PAGE and transferred onto polyvinylidene difluoride membranes (NTN Life Science Products). The blots were blocked with 5% (w/v) dried milk in PBS for 30 min, incubated with anti-D2-40 (Signet Laboratories) diluted 1:500 and then CD34 (Abcam) diluted 1:5000 in PBS containing 0.1% Tween 20. After rinsing in PBS, color was developed using an ECL system (Super Signal West Femto; Pierce Biotechnology Inc.). The bands were visualized using a diaminobenzidine solution containing 0.01% H_2_O_2_. The relative D2-40 signal intensity was obtained by dividing the intensity of D2-40 signals by that of GAPDH signals. Densitometric analysis of Western blot was performed using the Scion Image software (ver. Beta 4.02, Scion Corp., Frederick, MD). Data are expressed as means ± SEM. Statistical analyses were performed using one-way ANOVA followed by a post-hoc test (Fisher's PLSD) for multiple comparisons.

## Results

### Immunohistochemical findings for D2-40 and CD34

**Figure 1:**
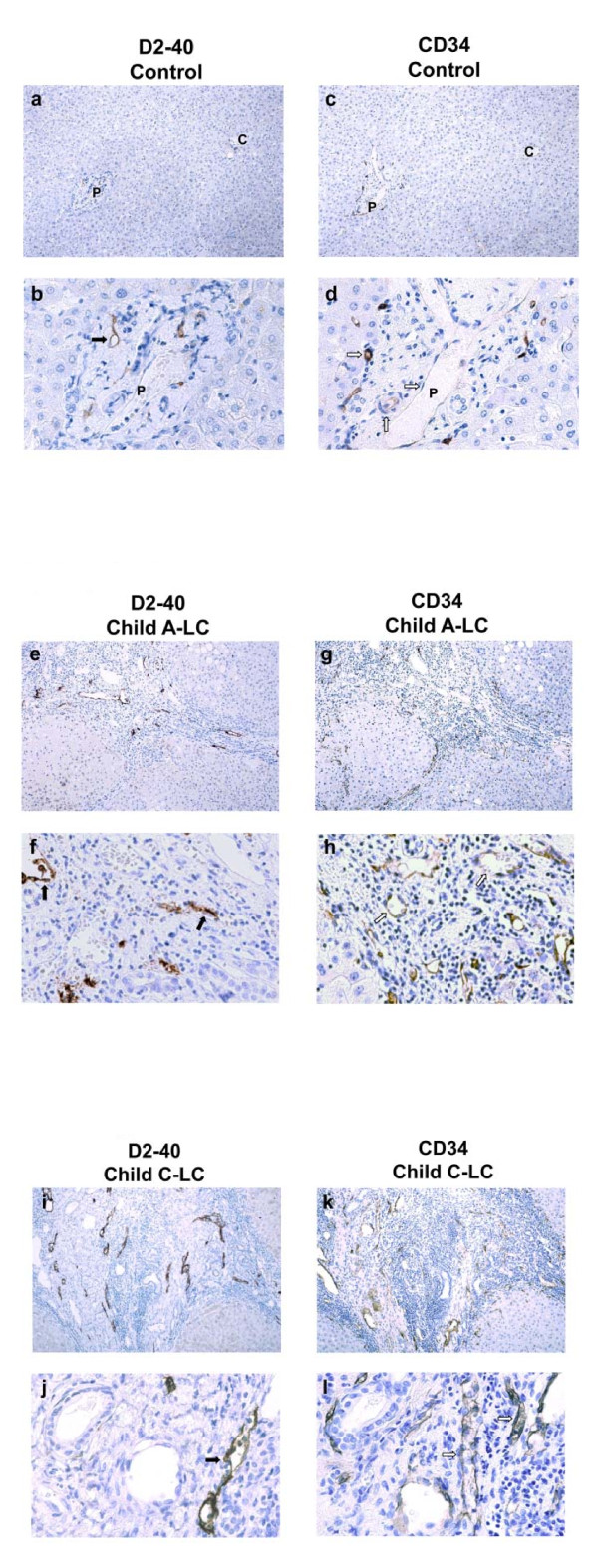
**Immunohistochemical studies of CD34 and D2-40**. Immunohistochemical staining with monoclonal antibodies D2-40 and CD34 was performed to examine lymphangiogenesis and angiogenesis in serial sections of liver samples obtained from normal portions of liver, Child-Pugh grade A cirrhotic liver (Child A-LC), and Child-Pugh grade C cirrhotic liver (Child C-LC). White arrows denote capillary or blood vessel endothelial cells. Black arrows denote lymph vessel endothelial cells. P denotes portal tracts or portal vein. C denotes central veins. **A**: Normal control liver tissue. Positive imunoreactivity of CD34 is observed in central vessels and portal vessels, as well as terminal portal venules. **a **and **b**: Lymphatic microvessels labeled by D2-40 immunostaining. **c **and **d**: Blood vessels labeled by CD34 immunostaining. White arrows show blood microvessels labeled by monoclonal antibody of CD34 only. Original magnification: **a**, **c**: ×100; **b**, **d **(portal tract): ×400: **B**: Child A-LC liver tissue. **e **and **f**: Lymphatic microvessels labeled by D2-40. **g **and **h**: Blood vessels labeled by CD34. CD34positive blood vessels are increased in cirrhotic liver. The number of D2-40-labeled lymphatic vessels in the fibrotic septa and the intensity of D2-40 imunoreactivity are increased in Child A-LC compared to the portal tract in normal control liver. Original magnification: **e**, **g**: ×100; **f**, **h **(fibrotic septa): ×400. **C**: Child C-LC liver tissue. **i **and **j**: Lymphatic microvessels labeled by D2-40. **k **and **l**: Blood vessels labeled by CD34. D2-40-positive lymphatic vessels are more abundant compared to control and Child A-LC liver tissues. Reaction products of CD34 are more abundantly located on hepatic sinusoids in regenerative hepatic nodules than in the parenchyma of control liver tissue. Original magnification: **i**, **k**: ×100; **j**, **l **(fibrotic septa): ×400.

In human control liver, lymph vessels were identified imunohistochemically as D2-40-positive and CD34-negative, while blood vessels were D2-40-negative and CD34-positive (Figures 1b and 1d). Positive imunoreactivity of CD34 was observed in central vessels and portal vessels, as well as terminal portal venules (Figures 1c and 1d). Slight imunoreactivity for D2-40 was found in the lymphatic vessels in the portal tract, some of which were parallel to the hepatic arteries. Most vessels were easily distinguishable as lymph vessels or blood vessels by a combination of D-2 40 and CD34 staining, because very few vessels were positive for both or negative for both markers. Liver parenchyma showed very few or no lymphatic vessels (Figures 1a and 1b).

**Figure 2:**
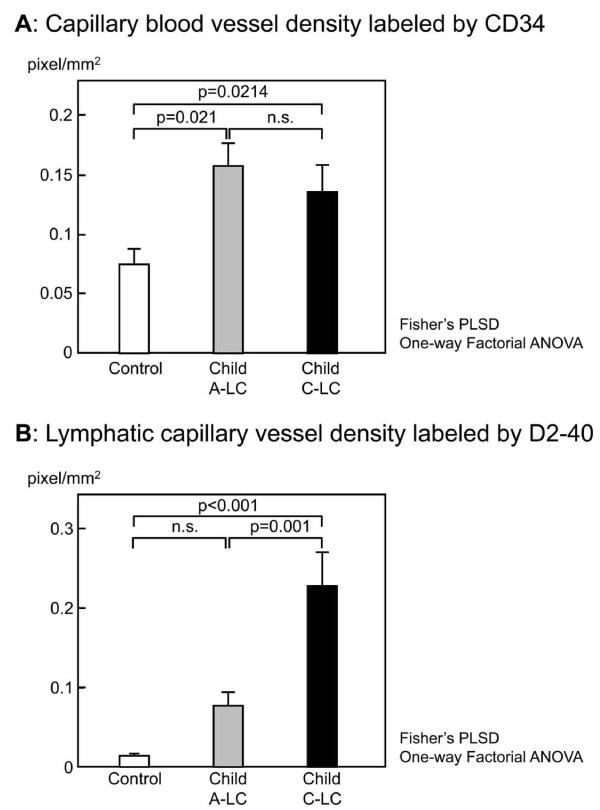
**Morphometric analyses of lymphatic vessels based on D2-40 immunostaining and vascular vessels based on CD34 immunostaining in control, Child A-LC, and Child C-LC liver samples**. Capillary blood vessel density (CBVD) and lymphatic capillary vessels density (LCVD) are expressed in pixels/sq mm. Data are mean ± SEM. **A**: CBVD of control, Child A-LC and Child C-LC the entire patient population (*n *= 28). Statistical analysis was performed using one-way ANOVA followed by Fisher's PLSD. **B**: LCVD of control, Child A-LC and Child C-LC liver samples. (*n *= 28). Statistical analysis was performed using one-way ANOVA, followed by Fisher's PLSD.

In Child A-LC liver, CD34-labeled blood vessels and D2-40-labeled lymphatic vessel in the fibrotic septa were more abundant compared to those in the portal tract in normal control liver (Figures 1e-h). Reaction products of CD34 were more abundant in regenerative nodules than in control liver (Figures 1g and 1h). Lymphatic vessels labeled by D2-40 appeared dilated and irregular in distribution (Figures 1e and 1f).

In Child C-LC liver, the D2-40-labeled lymphatic vessels were increased (Figures 1i and 1j). CD34 labeled blood vessels were more numerous than in control or Child C-LC liver (Figures 1k and 1l). Moreover, D2-40-positive lymphatic vessels were markedly denser and more variable in size than in Child A-LC liver.

### Morphometry of lymphatic vessels based on D2-40 immunostaining and blood vessels based on CD34 immunostaining

In the portal tract, CBVD evaluated by CD34 immunostaining were 0.0742 pixels/mm^3 ^in control, 0.1566 pixels/mm^3 ^in Child A-LC, and 0.1350 pixels/mm^3 ^in Child C-LC, and was significantly higher in Child C-LC (*p *= 0. 021) and Child A-LC (*p *< 0.021) than in control, although there was no significant difference between Child A-LC and Child C-LC (*p *= 0.09) (Figure 2). In the portal tract, LCVD evaluated by D2-40 immunostaining were 0.01459 pixels/mm^3 ^in control, 0.07692 pixels/mm^3 ^in Child A-LC and 0.229216 pixels/mm^3 ^in Child C-LC, and was significantly higher in Child C-LC than in Child A-LC (*p *= 0.001) or control (*p *< 0.001), and tended to be higher in control than in LC-A (*p *= 0.05) (Figure 2). Morphometry of lymphatic vessels based on D2-40 immunostaining showed a significant difference in of lymphatic vessel density between the mild cirrhotic stage and end-stage cirrhotic stage, and were prominently increased in the end-stage cirrhotic stage.

### Western blot for D2-40, CD34

**Figure 3:**
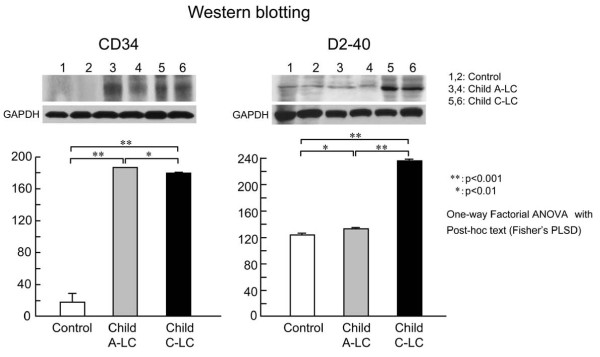
**Western blots for D2-40, CD34 protein expression**. CD34 and D2-40 protein expression in normal control, Child A-LC and Child C-LC liver samples. Representative Western blots are shown. Quantitative densitometric data from multiple samples (expressed as D2-40/GAPDH, *n *= 5 for each group) are shown as the mean value ± SEM. D2-40 protein level is high in Child C-LC liver samples and only moderate in control and Child A-LC liver samples, but CD34 protein level is equally high in Child A-LC and Child C-LV liver samples but low in control liver samples.

To confirm the immunohistochemical results, we investigated the protein expression of D2-40 and CD34 in control, Child A-LC and Child C-LC samples by Western blotting. Both CD34 and D2-40 expressions were found in control, Child A-LC and Child C-LC samples. Quantitative densitometric data (expressed as D2-40/GAPDH levels, *n *= 5 for each group) from multiple samples are shown as mean ± SEM. D2-40 protein was expressed abundantly in Child C-LC and only moderately in Child A-LC and control livers, but CD34 protein was expressed more abundantly in Child C-LC and Child A-LC than in control liver (Figure 3).

### Immunoelectron microscopic study

**Figure 4:**
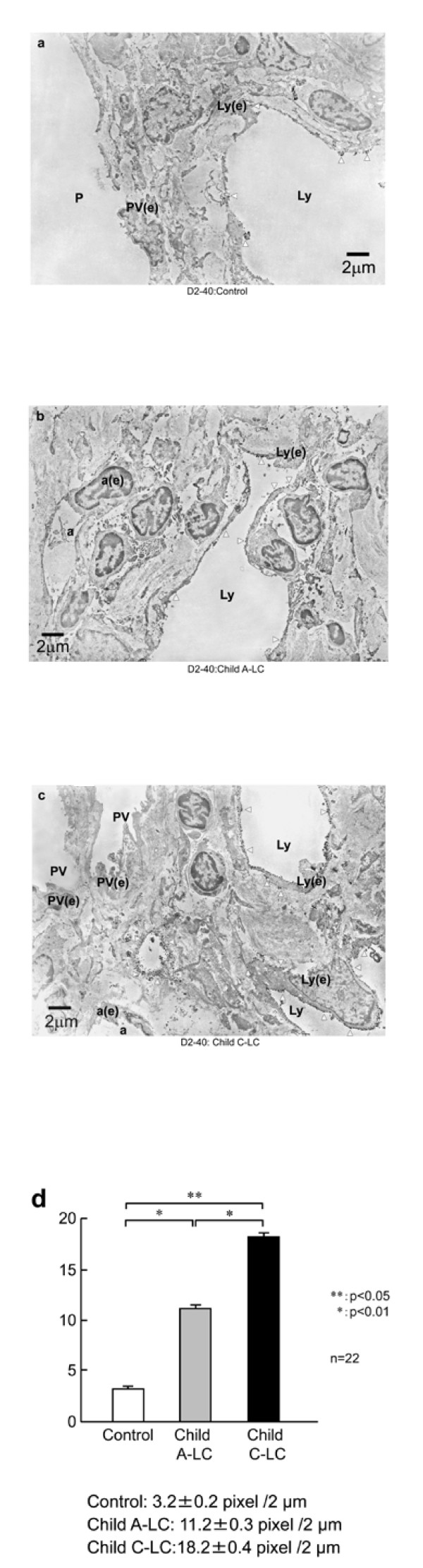
**Immunoelectron microscopic findings**. **a**: Distribution of D2-40 immunoreactivity at electron microscopic level in control liver tissue. Electron micrograph of immunogold-silver enhancement staining of a liver lymphatic vessel (Ly) in control liver sample. Localization of D2-40 is mainly on the plasma membrane of both luminal and abluminal surfaces of endothelial cells (arrowheads). **b**: Distribution of D2-40 immunoreactivity at electron microscopic level in Child A-LC liver tissue. Electron micrograph of immunogold-silver enhanced staining of a liver lymphatic vessel (Ly). **c**: Distribution of D2-40 immunoreactivity at electron microscopic level in Child C-LC liver tissue. D2-40 immunoreactivity is present along luminal and abluminal portions of the cell membrane (arrowheads) and is pronounced along cell processes. PV: portal venule; a: capillary artery; Ly: lymphatic vessel; P: portal vein, PV(e): portal vein, c(e): capillary arterial endothelial cell, and Ly(e): lymphatic endothelial cell. Scale bars: 2 μm. **d**: Morphometric analysis of immunogold-labeled D2-40. D2-40 labeling on lymphatic capillary endothelial cells is low in control liver tissue (3.2 ± 0.2/2 μm). In Child A-LC liver tissue, D2-40-1 labeling on lymphatic capillary endothelial cells (11.2 ± 0.3/2 μm) is significantly higher (*p *< 0.05) compared to control. In Child C-LC liver tissue, D2-40 labeling on lymphatic capillary endothelial cells (18.2 ± 0.4/2 μm) is significantly higher (*p *< 0.01) compared to control and also significantly higher (*p *< 0.05) compared to Child A-LC.

To clarify the ultrastructural localization of D2-40 and how expression is altered in cirrhotic liver, we conducted an immunoelectron microscopic study (Figure 4). In control liver tissue, electron-dense gold particles showing the presence of D2-40 were few and mainly localized on the luminal site of lymphatic capillary endothelial cells around the portal tract (Figure 4a).

In Child A-LC, electron dense gold particles showing the presence of D2-40 were increased compared to control liver, and were found not only on the luminal and abluminal regions of the proliferated lymphatic capillary endothelial cells (Figure 4b), but also on the basal sites of the cell membrane on the lymphatic capillary.

In Child C-LC samples, electron dense gold particles showing the presence of D2-40 were found on the proliferated lymphatic capillary ECs, and were more abundant than in either control or Child A-LC (Figure 4c). Moreover, gold particles demonstrating D2-40 were more abundant than in Child A-LC, not only on the luminal site, but also on basal sites of lymphatic capillary vessels (Figure 4c). Scanty immunostaining for D2-40 on hepatic sinusoids was observed at immunoelectron microscopic level in both control and cirrhotic liver tissues. Morphometric analysis of immunogold-labeled D2-40 (Figure 4d) revealed that D2-40 labeling was low in the lymphatic capillary endothelial cells of control liver tissue (3.2 ± 0.2/2 μm). D2-40- labeling in lymphatic capillary endothelial cells was significantly higher in Child A-LC (11.2 ± 0.3/2 μm) than in control (*p *< 0.05). D2-40 labeling in lymphatic capillary endothelial cells was significantly higher in Child C-LC, (18.2 ± 0.4/2 μm) than in control (*p *< 0.01) or Child A-LC (p < 0.05).

### Discussion and Conclusions

The present study demonstrated that D2-40 mAb is a selective marker of lymphatic endothelium in normal and cirrhotic liver tissues, and that this mAb does not react with the blood vessel endothelium, as demonstrated by the following results. First, D2-40 reacted with the endothelium of lymphatic channels, identified by morphological appearance, in normal and cirrhotic conditions. The endothelium of blood vessels including arteries, veins, and capillaries were not immunostained by D2-40 mAb. The sinusoids of normal liver showed scanty immunoreactivity. We confirmed that combined staining for D2-40 and CD34 is useful to distinguish between lymphatic microvessels and blood capillary vessels including hepatic arterial capillary and portal venules in human liver tissues. This is the first report to describe the analyses of the density of lymphatic vessels in liver cirrhosis.

In the present study, density of lymphatics differed significantly depending on the degree of liver fibrosis. Superficial lymphatic vessels are more clearly visible in cirrhosis, and the lymph vessels appear to be enlarged when observed under a scanning electron microscope [7]. Our histochemical results showed that intrahepatic lymph vessels, i.e., non-superficial lymph vessels, are also enlarged in patients with liver cirrhosis, especially in those who had end-stage cirrhosis undergoing liver transplantation (Child C-LC). This phenomenon is attributable to increased lymph production, which results from the impaired drainage of vascular flow from the sinusoid to the central or terminal hepatic veins associated with lobular distortion in patients with advanced liver disease or cirrhosis. The most common cause of ascites in cirrhosis is elevated pressure in the hepatic circulation (portal hypertension). The elevated pressure causes leakage of fluid from the lymph vessels in and around the liver into the abdominal cavity. The role of lymphatic abnormalities on the formation or reabsorption of ascites should be examined in further studies.

A previous study revealed that the number (density) of blood vessels in the portal tract is highest during the stage of mild fibrosis and that the area in the portal tract is greater in normal liver and mild fibrosis than in moderate and severe fibrosis [10]. These data indicate that neither the number nor area of blood vessels increase concomitantly with the portal area enlargement. These changes in number and area of blood vessels differ from those seen in lymphatic vessels [10]. The observation in experimental animals of a strong negative correlation between density of the lymphatic network in the sinusoid and trans-sinusoidal macromolecular exchange suggests that increased lymph flow is an important mechanism by which fluid can bypass increased sinusoidal/postsinusoidal resistance [5]. Furthermore, the lymphatic network has been reported to be enlarged according to fibrotic changes in cirrhotic rats [5]. Our results of immunohistochemical and morphometric analyses of human liver samples are in agreement with the findings of previous findings, demonstrating that lymphatic vessels in the liver increase in size and number with the progression of chronic fibrosis, and are especially increased in end-stage cirrhosis necessitating transplantation. The changes in size and number of the lymph vessels differ considerably from those seen in blood vessels. Several growth factors, such as vascular endothelial growth factor (VEGF)-C are involved in lymphatic angiogenesis [23]. These factors promote the proliferation of lymphatic endothelial cells and formation of lymphatic vessels.

In an *in vitro *study using primary human lung microvascular lymphatic endothelial cells, small interfering RNA (siRNA) mediated silence of podoplanin gene expression has the dramatic effect of blocking capillary tube formation in Matrigel, which clearly demonstrates that early activation of RhoA in the lymphangiogenic process -required for successful establishment of the capillary network- is dependent on podoplanin expression [24]. The data obtained in the present study suggest that the factors associated with increased density of lymphatic and blood vessels might be different. Although we did not investigate the mechanisms of dilatation and proliferation of lymph vessels in different stages of cirrhosis, these changes are thought to result from the disturbance of microcirculation associated with liver fibrosis and lobular distortion.

Regarding lymphatic endothelial cell marker, LYVE-1 reactivity is marked in cirrhotic liver, and is expressed also on hepatic sinusoidal endothelial cell, especially in normal liver tissues [13]. However, some investigators have questioned the specificity of LYVE-1 for lymphatic endothelial cells [13]. This study showed scanty D2-40 immunostaining on sinusoids at immunoelectron microscopic level in both control and cirrhotic tissues. A previous study using corrosion casting/scanning electron microscopy showed that the portal lymphatic networks develop around the portal triads, and extend distally as far as the terminal portal tract [25]. No tendency for the portal lymphatic vessels to run preferentially along the interlobular arteries, veins, or bile ducts was observed. These findings apparently support the hypothesis that the portal lymph comes mainly from the hepatic sinusoids.

It has long been a puzzle of how fluid in the space of Disse reaches the lymphatics in the portal tract. The limiting plate of heptocytes possesses many pores (1-3 μm in diameter) that open to the space of Mall. The pores are continuous with the channels between hepatocytes and open into the space of Disse. The space of Disse is connected through these channels to the space of Mall, and then drains through the interstitial space of the portal tracts to enter interlobular lymphatics [26]. Lymph vessels and branches of the portal vein are readily discerned in the portal tracts. A marked increase in the number of lymph vessels was noted in the portal tract of idiopathic portal hypertension and primary biliary cirrhosis compared to that of the control liver [27,28]. Advanced cirrhosis is thought to result from increased lymph production, which in turn arises from disturbance of the microcirculation associated with portal hypertension.

In the present study, D2-40 was mainly localized in lymphatic endothelial cells and the expression in the regenerative nodules became stronger as cirrhosis advanced. At immunoelectron microscopic level, we were able to distinguish between the portal venule, capillary artery, and lymphatic endothelial cells.

A limitation of the present study is that we investigated a small number of samples (five in each group), which may be a disadvantage in the analysis and comparison of vessel density between study groups. A larger number of samples should be examined to confirm the present finding.

## Competing interests

The authors declare that they have no competing interests.

## Authors' contributions

HY conceptualized and designed the study, analyzed and interpreted the data and wrote the manuscript. HY and MO collected the clinical materials. HY and KY performed immunohistochemisty, electron microscopy, and Western blots. HY, KY, FK, MO, and TH assisted in the analysis and interpretation of data. HY supervised the research and edited the manuscript. All authors critically reviewed the manuscript, and read and approved the final version of the manuscript.

## Pre-publication history

The pre-publication history for this paper can be accessed here:

http://www.biomedcentral.com/1471-230X/10/131/prepub
